# Functional Mining of the *Crotalus* Spp. Venom Protease Repertoire Reveals Potential for Chronic Wound Therapeutics

**DOI:** 10.3390/molecules25153401

**Published:** 2020-07-28

**Authors:** David Meléndez-Martínez, Luis Fernando Plenge-Tellechea, Ana Gatica-Colima, Martha Sandra Cruz-Pérez, José Manuel Aguilar-Yáñez, Cuauhtémoc Licona-Cassani

**Affiliations:** 1 Tecnologico de Monterrey, Escuela de Ingeniería y Ciencias. Av. Eugenio Garza Sada 2501 sur, Monterrey, N.L. 64849, Mexico; a00824975@itesm.mx (D.M.-M.); aguilar.manuel@tec.mx (J.M.A.-Y.); 2 Departamento de Ciencias Químico-Biológicas, Instituto de Ciencias Biomédicas, Universidad Autónoma de Ciudad Juárez, Anillo Envolvente del PRONAF y Estocolmo s/n, Ciudad Juárez, Chih. 32310, Mexico; fplenge@uacj.mx (L.F.P.-T.); agatica@uacj.mx (A.G.-C.); 3 Herpetario de la Universidad Autónoma de Querétaro, Facultad de Ciencias Naturales, Universidad Autónoma de Querétaro, Juriquilla, Qro. 76230, Mexico; martha.sandra.cruz@uaq.mx; 4 Scicore Medical SAPI de CV, 2612-13 Alfonso Reyes Av. Del Paseo Residencial, Monterrey, N.L. 64920, Mexico

**Keywords:** *Crotalus*, metalloproteases, rattlesnakes, serine proteases, snake venom, wound healing

## Abstract

Chronic wounds are a major health problem that cause millions of dollars in expenses every year. Among all the treatments used, active wound treatments such as enzymatic treatments represent a cheaper and specific option with a fast growth category in the market. In particular, bacterial and plant proteases have been employed due to their homology to human proteases, which drive the normal wound healing process. However, the use of these proteases has demonstrated results with low reproducibility. Therefore, alternative sources of proteases such as snake venom have been proposed. Here, we performed a functional mining of proteases from rattlesnakes (*Crotalus ornatus*, *C. molossus nigrescens*, *C. scutulatus,* and *C. atrox*) due to their high protease predominance and similarity to native proteases. To characterize *Crotalus* spp. Proteases, we performed different protease assays to measure and confirm the presence of metalloproteases and serine proteases, such as the universal protease assay and zymography, using several substrates such as gelatin, casein, hemoglobin, L-TAME, fibrinogen, and fibrin. We found that all our venom extracts degraded casein, gelatin, L-TAME, fibrinogen, and fibrin, but not hemoglobin. *Crotalus ornatus* and *C. m. nigrescens* extracts were the most proteolytic venoms among the samples. Particularly, *C. ornatus* predominantly possessed low molecular weight proteases (P-I metalloproteases). Our results demonstrated the presence of metalloproteases capable of degrading gelatin (a collagen derivative) and fibrin clots, whereas serine proteases were capable of degrading fibrinogen-generating fibrin clots, mimicking thrombin activity. Moreover, we demonstrated that *Crotalus* spp. are a valuable source of proteases that can aid chronic wound-healing treatments.

## 1. Introduction

Chronic wounds are interruptions in the epithelial surface that endogenous resources fail to repair during a normal period of time [1]. Overall, chronic wounds including diabetic, venous, and pressure ulcers affect more than 6 million people and account for an annual public expense of almost 10 billion dollars in the USA alone [2,3,4]. While most chronic wound treatments are related to wound dressings [5,6], active wound treatments such as enzymes [7,8], skin grafts [9], and growth factors [10] are emerging with great potential as they enhance the biological healing process.

The wound healing biological process includes the combined action of proteases (e.g., thrombin and matrix metalloproteases, MMPs) along several overlapping steps, namely hemostasis, inflammation, cell migration and proliferation, and skin remodeling [11]. Proteases of plant (papain from *Carica papaya* [12] and bromelain from *Ananas comosus* [13]) and microbial origins (collagenase from *Clostridium histolyticum* or vibriolysin from *Vibrio protelyticus*) were the first enzymes used as wound healing active therapeutics to debride the necrotic tissue, diminish inflammation, and increase angiogenesis. However, these enzymes showed low reproducibility in terms of the patients’ healing outcomes [14]. Alternative sources of proteases that may provide improved results to expedite chronic wound healing include maggot digestive excretions [15,16], fish epithelial mucus [17,18,19], and snake venoms [20,21,22,23]. Snake venom proteases from the *Crotalus* genus are of particular interest for exploration due to their greater proteolytic activity in comparison to other venomous snakes [24].

Some snake venoms are specialized to generate the disruption of fundamental homeostatic processes. This venom specialization confers to the toxins a high affinity to a particular target, which converts each toxin into a potential source for design and development of new drugs [25]. For this purpose, rattlesnakes (*Crotalus* sp.) is the most representative genus of venomous snakes with 51 species described [26], 42 of which are distributed in Mexico [27]. *Crotalus* sp. venom contains many toxins, several of which are snake venom metalloproteases (SVMP) and snake venom serine proteases (SVSP). Together, both protease families represent about the 45% of the toxin abundance in their venoms and can be as high as 93% (*Crotalus tigris*) [28]. Some SVMPs and SVSPs are used as anticoagulant agents to treat ischemic strokes [29], peripheral arterial occlusions [30], and acute cerebral infarction [31] due to their capacity to degrade the extracellular matrix and hemostasis-related proteins, such as collagens, fibrinogen, and fibrin [32,33,34]. More importantly, due to their similarity to human MMPs [35], snake venom proteases are potential wound-healing therapeutics as they can participate as procoagulants/platelet aggregators [36,37,38,39,40], inflammation modulators [41,42,43,44], cell migration stimulators, cell proliferators [45,46], skin fibroblasts activators [47], cell migration factors [48,49], angiogenesis enhancers [36,38], and activators of MMP-2 and MMP-9 [50,51,52].

Here, we explore the potential of the protease activity of four different rattlesnakes with specific interest in their potential application in wound-healing therapeutics. Venoms of *Crotalus ornatus*, *C. molossus nigrescens*, *C. scutulatus,* and *C. atrox* specimens from the Chihuahuan Desert and Mexican plateau were extracted for testing. Protease characterization was performed using different quantitative and qualitative enzymatic assays including the universal proteolytic assay and zymography and with specific substrates, such as gelatin, L-TAME, fibrinogen, and fibrin. Using these techniques, we found that the venoms used in this study, specifically *C. ornatus* and *C. m. nigrescens*, were rich in protease activity. The results reported here indicate the first step towards a potential for *Crotalus* snake venom proteases for the application as suitable wound-healing therapeutics.

## 2. Results

### 2.1. Crotalus Spp.’ Venom Toxin Families Identification by SDS-PAGE

**Figure 1:**
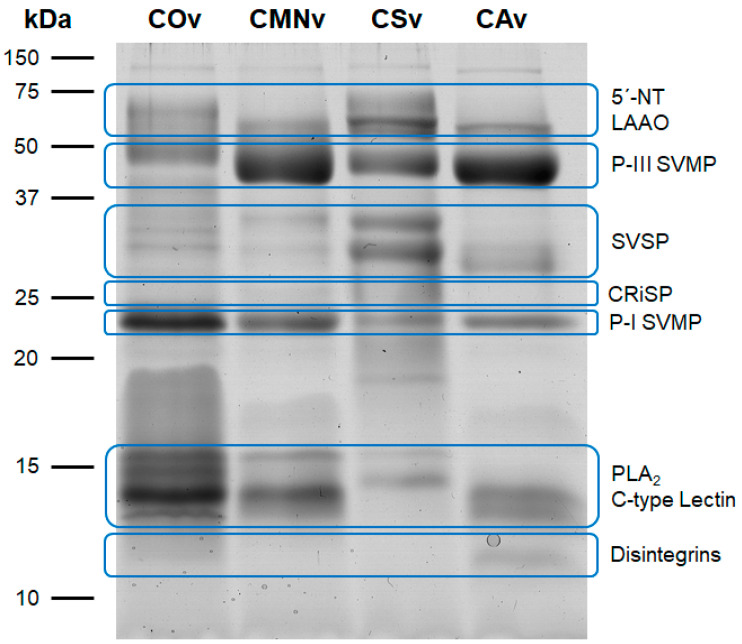
SDS-PAGE venom banding profile for *C. ornatus* (COv), *C. m. nigrescens* (CMNv), *C. scutulatus* (CSv), and *C. atrox* (CAv). 15 µg of each venom were separated on a 12% SDS-PAGE and stained with Coomassie colloidal stain. Blue ovals enclose the typical molecular weight intervals for the most abundant toxin families according to Mackessy [53]. 5′-NT: 5’-nucleotidases; CRiSP: cysteine-rich secreted proteins; LAAO: L-amino acid oxidases; PLA_2_: phospholipase A_2_; SVMP: snake venom metalloproteases; SVSP: snake venom serine proteases.

First, we performed 12% SDS-PAGE with 15 µg of each venom sample in order to explore the venom protein profile. Venom banding patterns for *Crotalus ornatus*, *C. m. nigrescens*, *C. scutulatus,* and *C. atrox* showed protein molecular weights from 12 to 116 kDa (Figure 1). All venoms tested contained similar protein diversity at least through visual inspection of the 12% SDS-PAGE. *Crotalus ornatus* venom was separated into 14 bands with different molecular weights (116, 75, 64, 52, 40, 31, 28, 23, 19, 16, 13.8, 13, 12.5, and 12 kDa). Very similar band profiles were observed for *C. m. nigrescens* (116, 74, 70, 65, 46, 33, 27, 22, 19, 17, 14, 13, 12.7 and 12 kDa) and *C. atrox* (106, 63, 59, 46, 29, 27, 24, 17, 14, 12.7, 12, and 11 kDa) with 12 protein bands. Lastly, *C. scutulatus* displayed 13 bands of similar molecular weights (106, 74, 68, 58, 48, 33, 27, 24, 19, 17, 16, 13, and 12 kDa).

Additionally, we identified typical toxin families described previously [53] in all the venoms we analyzed (Figure 1, blue ovals). In *C. ornatus* venom, we found a P-I SVMP and PLA_2_/C-type Lectin bands predominance, whereas *C. m. nigrescens*, *C. scutulatus,* and *C. atrox* venoms had a higher abundance of the P-III SVMP band. Finally, *C. scutulatus* venom showed a greater expression of SVSP and 5’-NT/LAAO bands in comparison to the other venom samples.

### 2.2. Protease Inhibitors 

**Figure 2:**
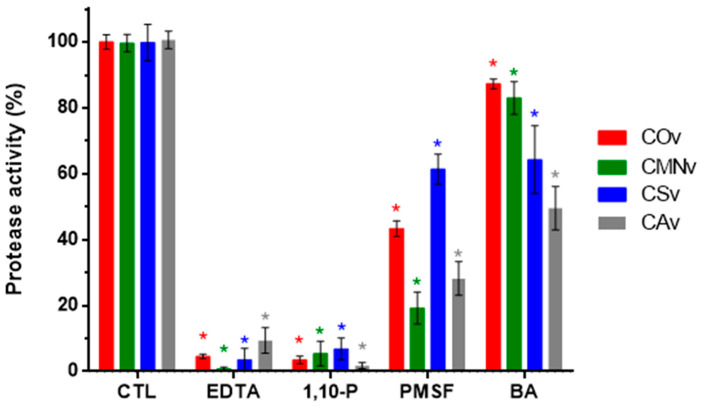
Inhibitor modulation of protease activity for *C. ornatus* (COv), *C. m. nigrescens* (CMNv), *C. scutulatus* (CSv), and *C. atrox* (CAv). 10 µg of each venom were preincubated without inhibitor (control, CTL) or with 5 mM of metalloprotease inhibitors (EDTA and 1,10-P) and serine protease inhibitors (PMSF and BA) for 10 min at 37 °C before substrate addition. Enzymatic activity is expressed in protease activity percentage in comparison to CTL. Data are presented as the mean of at least three independent experiments with its respective standard error, statistically significant difference (*p* < 0.05) with respect to control for each venom is represented with an asterisk (*).

In order to confirm the nature of the proteases expressed in each venom, we performed universal protease activity assays using casein as the substrate together with several protease inhibitors, namely, EDTA, 1,10-P, PMSF, and BA (Figure 2). All venoms tested in this assay were mostly inhibited by metalloprotease inhibitors (EDTA and 1,10-P), with final protease activity below 10%. For EDTA: *C. ornatus*, 4.6%; *C. m. nigrescens*, 0.7%; *C. scutulatus,* 3.5%; and *C. atrox*, 9.4%. For 1,10-P: *C. ornatus*, 3.5%; *C. m. nigrescens*, 5.4%; *C. scutulatus,* 6.8%; and *C. atrox*, 1.7%.

Regarding serine protease inhibitors (PMSF and BA), *C. m. nigrescens* and *C. atrox* showed higher inhibition with PMSF with a remaining protease activity of 19.2% and 28.3%, respectively. BA inhibition was higher in *C. scutulatus* and *C. atrox* venoms with a remaining protease activity of 64.3% and 49.5%, respectively.

### 2.3. In-Gel Zymography

**Figure 3:**
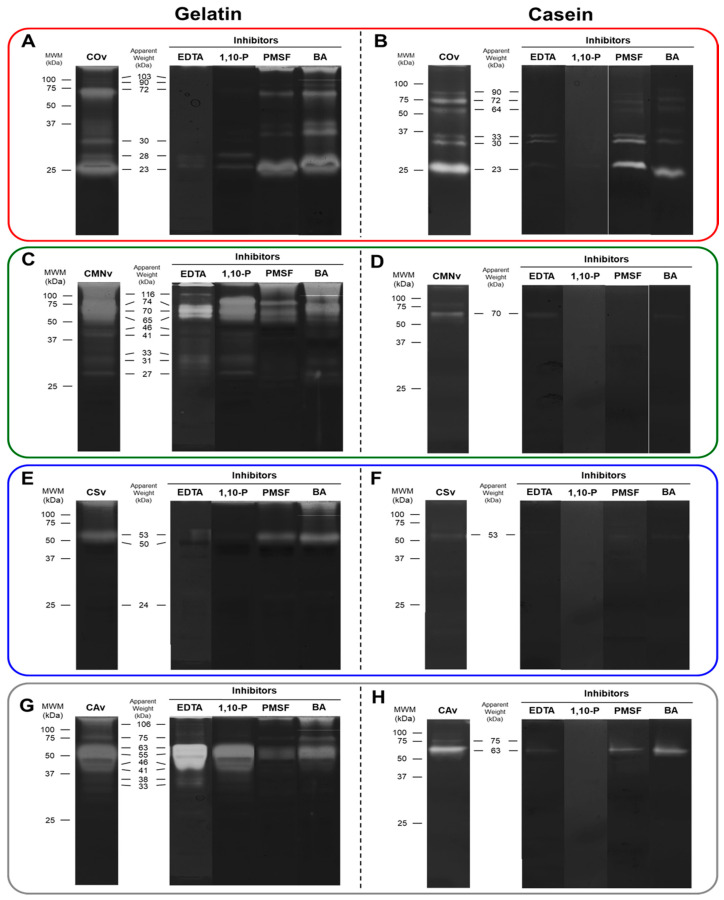
In-gel zymography for proteases of venoms and their inhibitors. 8 µg of (**A**,**B**) *C. ornatus* (red box), (**C**,**D**) *C. m. nigrescens* (green box), (**E**,**F**) *C. scutulatus* (blue box), and (**G**,**H**) *C. atrox* (gray box) venoms were separated on a 12% SDS-PAGE copolymerized with 1% (*w/v*) gelatin (**A**,**C**,**E**,**G**) or casein (**B**,**D**,**F**,**H**). Each venom protease activity was tested versus 5 mM protease inhibitors: EDTA, 1,10-P, PMSF, and BA. As a control for each sample, the venom was incubated with no inhibitor. Apparent molecular weights (kDa) for the bands were computed with a GelAnalyzer and are denoted in the figure for each venom.

In order to identify the molecular weight of all the snake venom metalloproteases and serine proteases, we performed in-gel zymography using gelatin and casein together with the previously used protease inhibitors (Figure 3). We performed a combinatorial screening of three substrate concentrations (0.5, 1, and 1.5%) of each substrate with seven different venom concentrations (1, 2, 4, 6, 8, and 10 µg) and found that better contrast was observed for the pair 1% substate–8 µg of venom (Figure S1). In general, we found that gelatin zymography demonstrated more active bands than did casein zymography in all the venoms, and that *C. ornatus* have a protease profile characterized mostly by low molecular weight proteases (23–33 kDa) in comparation to the other venom extracts that presented more activity on high molecular weight proteases (>45 kDa).

In order to identify the molecular weight of all the snake venom metalloproteases and serine proteases, we performed in-gel zymography using gelatin and casein together with the previously used protease inhibitors (Figure 3). We performed a combinatorial screening of three substrate concentrations (0.5, 1, and 1.5%) of each substrate with seven different venom concentrations (1, 2, 4, 6, 8, and 10 µg) and found that better contrast was observed for the pair 1% substate–8 µg of venom (Figure S1). In general, we found that gelatin zymography demonstrated more active bands than did casein zymography in all the venoms, and that *C. ornatus* have a protease profile characterized mostly by low molecular weight proteases (23–33 kDa) in comparation to the other venom extracts that presented more activity on high molecular weight proteases (>45 kDa).

*Crotalus ornatus* venom showed six active bands in both gelatin and casein zymographies (Figure 3A,B). A 103 kDa band was detected only on gelatin zymography and 64 kDa band was specific to casein zymography. For gelatin zymography, 90, 28, and 23 kDa bands were only inhibited by metalloprotease inhibitors (EDTA and 1,10-P) and 103, 72, and 30 kDa bands were modulated by all the inhibitors tested. In casein zymography, all bands were inhibited by 1,10-P. The 90, 72, and 64 kDa bands were inhibited by EDTA, and the same bands were slightly inhibited by serine protease inhibitors. *Crotalus m. nigrescens* venom showed nine active bands on gelatin zymography (Figure 3C), whereas in casein zymography, only one band (70 kDa) was detected (Figure 3D). For gelatin zymography, 74, 46, and 41 kDa bands were only inhibited by metalloprotease inhibitors, 33, 31, and 27 kDa bands were inhibited by serine protease inhibitors, 116, 70, and 65 kDa bands were modulated by all the inhibitors. The casein zymography band was fully inhibited by 1,10-P, PMSF, and BA. *Crotalus scutulatus* venom showed the least diversity of proteases with only three bands on gelatin zymography (Figure 3E) and one on casein zymography (Figure 3F). All bands detected on both zymography substrates were inhibited by metalloprotease inhibitors. Finally, *Crotalus atrox* venom showed eight proteolytic bands on gelatin zymography (Figure 3G) and two bands (75 and 63 kDa) in casein zymography (Figure 3D). From those, only the 106 kDa band was inhibited by metalloprotease inhibitors, the 55 and 46 kDa bands were inhibited by serine protease inhibitors, the 41–33 kDa bands were modulated by all the inhibitors except EDTA. The 75 kDa band was only inhibited by 1,10-P on both zymography substrates.

### 2.4. Crotalus Ornatus Venom Proteases are the Most Active among Crotalus Spp.’ Venoms

**Figure 4:**
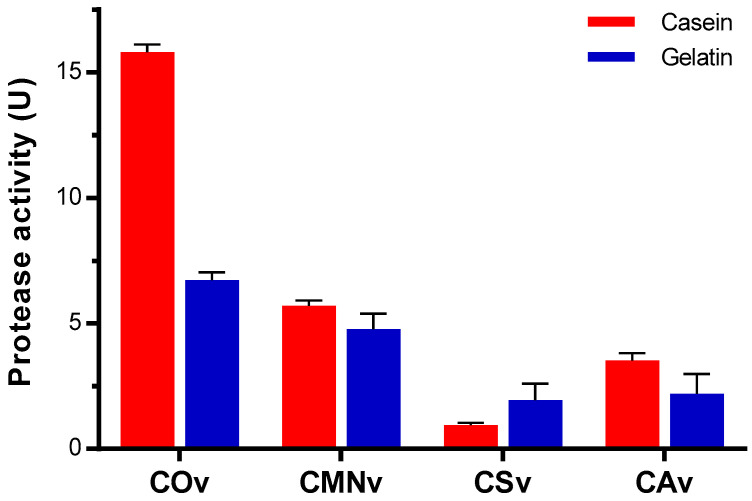
Proteolytic activity for *Crotalus* venoms using different substrates. 10 µg of venom from *C. ornatus* (COv), *C. m. nigrescens* (CMNv), *C. scutulatus* (CSv), and *C. atrox* (CAv) were incubated with 1% (*w/v*) substrate solution (casein or gelatin) for 1 h at 37 °C. Enzymatic activity is expressed in protease activity units (mmol equivalent of L-leucine formed per minute per mg of protein). Data are presented as the mean of at least three independent experiments with its respective standard error.

We performed protease assays with casein, gelatin, and hemoglobin. As previously suggested by in-gel zymography experiments, we detected a substrate bias for all venoms tested towards gelatin. In general, *C. ornatus* venom showed 3 to 15 times higher activity compared to the rest of the venom extracts when casein was used as a substrate (Figure 4). Regarding gelatin, all venoms had lower proteolytic activity, with *C. ornatus* and *C. m. nigrescens* being the most gelatinolytic venoms. During our assays, no significant proteolyzed hemoglobin was observed.

### 2.5. Snake Venom Serine Protease Characterization on Crotalus Spp.’ Venoms 

**Figure 5:**
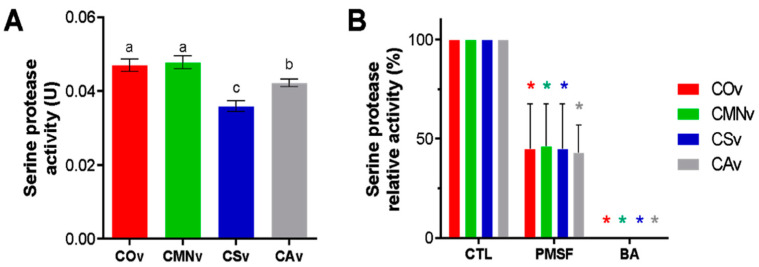
Serine protease activity for *Crotalus* venoms using L-TAME and inhibitor modulation. (**A**) Serine protease activity for *C. ornatus* (COv), *C. m. nigrescens* (CMNv), *C. scutulatus* (CSv), and *C. atrox* (CAv). 5 µg of each venom were incubated with 1.0 mM L-TAME. Enzymatic activity is expressed in serine protease activity units. Statistical difference between venom samples is denoted with different letters (*p* < 0.05). (**B**) Inhibitor modulation in serine protease activity for all venoms. 5 µg of each venom were preincubated with 5 mM PMSF or EDTA for 10 min before substrate addition. Enzymatic activity is expressed in protease activity percentage in comparison to CTL. Data are presented as the mean of at least three independent experiments with its respective standard error, statistically significant difference (*p* < 0.05) with respect to control for each venom is represented with an asterisk (*).

In order to quantify the serine protease activity, we performed the esterase assay for each venom extract using L-TAME as the substrate. We observed esterase activity in all the venom samples tested (Figure 5A). *Crotalus ornatus* venom and *C. m. nigrescens* venom extracts showed more activity than did *C. scutulatus* and *C. atrox* venom extracts. On the other hand (Figure 5B), all the venoms were inhibited by PMSF and to a greater extent BA.

### 2.6. Snake Venom Metalloproteases and Snake Venom Serine Proteases

**Figure 6:**
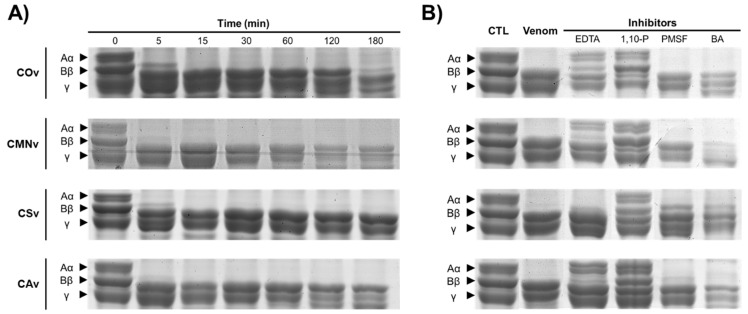
Fibrinogenolytic activity for *Crotalus* venoms. (**A**) Fibrinogenolytic activity for *C. ornatus* (COv), *C. m. nigrescens* (CMNv), *C. scutulatus* (CSv), and *C. atrox* (CAv) at different time intervals (0–180 min). (**B**) Inhibitor modulation in fibrinogenolytic activity for all venoms, each reaction was preincubated with 5 mM protease inhibitors: EDTA, 1,10-P, PMSF, and BA, then the venom-inhibitor mixtures were incubated with fibrinogen for 60 min. Samples (15 µg) were separated on a 10% SDS-PAGE and stained with Coomassie colloidal staining to detect the degradation of fibrinogen Aα, Bβ, and γ chains.

Fibrinogenolytic activity of the venom extracts was studied by exposing human fibrinogen to all the venoms, measuring the time-dependent degradation and the effect of the protease inhibitors over the fibrinogen (Figure 6). All the venoms degraded the Aα fibrinogen after 5 min of exposition and when the incubation time was increased, the Bβ fibrinogen chain was degraded by all the venoms (Figure 6A). From all venom samples, *C. m. nigrescens* and *C. atrox* venoms were the more fibrinogenolytic extracts.

The effect of the protease inhibitors in the fibrinogenolytic activity was similar in all venom extracts (Figure 6B). EDTA and 1,10-P (metalloprotease inhibitors) caused partial degradation of Aα and Bβ fibrinogen chains. Particularly, we observed that when SVMP were inhibited by 1,10-P, SVSP caused coagulation of the fibrinogen. On the other hand, serine protease inhibition (PMSF and BA treatments) caused a higher degradation of Aα and Bβ fibrinogen chains in comparation to that of control.

### 2.7. Crotalus Sp.’ Snake Venom Metalloproteases Degrade Fibrin

**Figure 7:**
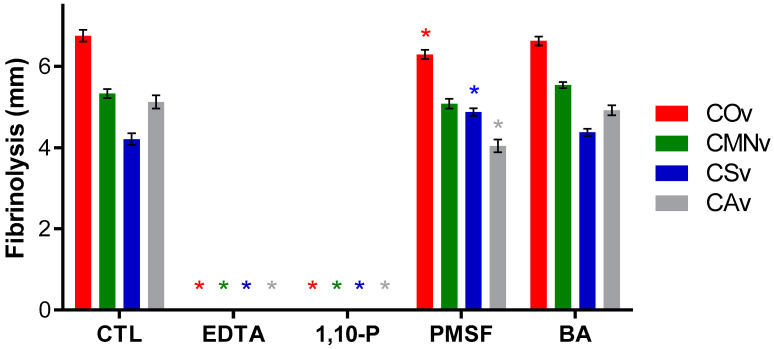
Fibrinolytic activity for *Crotalus* venoms. 10 µg of *C. ornatus* (COv), *C. m. nigrescens* (CMNv), *C. scutulatus* (CSv), and *C. atrox* (CAv) venoms were preincubated without inhibitor (CTL) or with 5 mM of each inhibitor: EDTA, 1,10-P, PMSF, or BA, for 10 min at 37 °C prior to the assay. Fibrinolytic activity is presented as the mean of at least three independent experiments with its respective standard error, statistically significant difference (*p* < 0.05) with respect to control for each venom is represented with an asterisk (*).

The fibrinolytic activity of each venom extract was studied by a standard enzymatic assay using additional protease inhibitors. All venom extracts degraded fibrin (Figure 7). *Crotalus ornatus* venom was the most fibrinolytic extract whereas *C. scutulatus* showed the lowest activity against fibrin. On the other hand, EDTA and 1,10-P (metalloprotease inhibitors) were effective in the inhibition of fibrin proteolysis whereas PMSF and BA (serine protease inhibitors) did not show any effect on this proteolytic activity.

## 3. Discussion

Snake venoms are animal secretions containing a huge diversity of molecules with a broad medical applicability [54]. Toxins represent a source of potential new drugs for diverse medical problems such as cancer, chronic pain, and neurological disorders [55]. While protein mining and bioactivity tests are still required, the results here shown represent the first step towards exploring the applicability of *Crotalus* spp.-derived venoms as therapeutics for the chronic wound healing industry. For readability, we divided the discussion according to protein and protease profiling, identification, and substrate.

### 3.1. Venom Profiling

*Crotalus ornatus* venom is mostly characterized for its capacity for hemorrhage formation [56] and hemostatic alteration, such as fibrinolysis and fibrinogenolysis [57]. Thus, most of the toxin description in this venom is related to SVMP isolation, all within a molecular weight range of 23–28 kDa. From those, three hemorrhagic P-I SVMP have been described. First, Sánchez et al. [58] characterized CMM4, a fibrinolytic and hemorrhagic P-I SVMP, with a molecular mass range between 23 and 26 kDa and a pI of 11.3. Second, Chen and Rael [59] characterized M5, a fibrinolytic, fibrinogenolytic and hemorrhagic P-I SVMP, with a molecular mass of 25 kDa and a pI of 7.6. Third, Rael et al. [60] described M4, a non-hemorrhagic fibrinolytic and fibrinogenolytic P-I SVMP, with a molecular mass of 27 kDa and a pI of 9.6. These three toxins must have been present in our venom sample and observed as the 28 and 23 kDa bands. Finally, Tsai et al. [61] found PLA_2_ with a molecular weight range between 11–14 kDa, in which range four bands were detected in our sample.

*Crotalus m. nigrescens* venom is described to be hemotoxic and hemorrhagic [62] and produces fibrinolysis and fibrinogenolysis [63]. From this venom, only two toxins have been isolated and characterized, Ramírez et al. [63] purified proteinase E, a 21.39 kDa P-I SVMP, and a 75 kDa thrombin-like SVSP (TL-SVSP). In our sample, we obtained bands with similar molecular weight (22 and 74 kDa). Also, Borja et al. [64] demonstrated the presence of SVMP, SVSP, and PLA_2_ using polyclonal antibodies from rabbits immunized with *C. simus* venom, they reported two SVSPs of 33 and 75 kDa, several SVMP bands of 64 and 37–20 kDa, and a 13 kDa PLA_2_ band, all of these bands are displayed in our sample of *C. m. nigrescens* venom. It is worth noting that the venom used in that experiment may have been extracted from adult specimens, due to its high concentration of SVMP and lack of myotoxins.

*Crotalus scutulatus* venom have different phenotypes depending on the distribution of the specimens: type A, type B, or type A + B. The venom used in this study belonged to the type B phenotype. Massey et al. [65] and Dobson et al. [66] characterized this venom and showed that the bands from 12 to 14 kDa belong to PLA_2_, 33 kDa to SVSP, and 48 and 68 kDa to SVMP, the 23 kDa band can contain SVMP or CRiSP. Also, Dobson et al. [66] mentioned that this venom contains two kallikrein-like SVSPs (KL-SVSP) between 37 and 25 kDa; both bands were observed in our sample with 33 and 27 kDa. Two isolated PLA_2_ were characterized by Zepeda et al. [67] and demonstrated the existence of two isoforms of 14.5 and 14.4 kDa with pI of 9.2 and 7.4, respectively. Also, a 27 kDa (pI 4.7) hemorrhagic and fibrinogenolytic P-I SVMP was isolated [68]. In our sample, we observed a band with the same molecular weight.

*Crotalus atrox* venom has been previously described by Calvete et al. [69] using proteome analysis. In that study, the authors identified that the majority of its toxins belong to SVMPs (49.7%) and SVSPs (19.8%). In our samples, we identified that the 12–14 kDa bands correspond to PLA_2_, 17 kDa band to C-type lectin, 24 and 27 kDa bands to SVMP or SVSP, 46 kDa band to SVMP, 59 kDa to SVMP or LAAO, and 106 kDa band to SVMP. In addition, Bjarnason et al. [70] described two KL- SVSPs named EI and EII, with molecular weights of 27.5 and 29.2 kDa, respectively. Both bands were present in our venom sample. The same author isolated two hemorrhagic 24 kDa P-I SVMPs, named Ht-c and Ht-d [71]. A band with this molecular mass was observed in this *C. atrox* extract.

**Figure 8:**
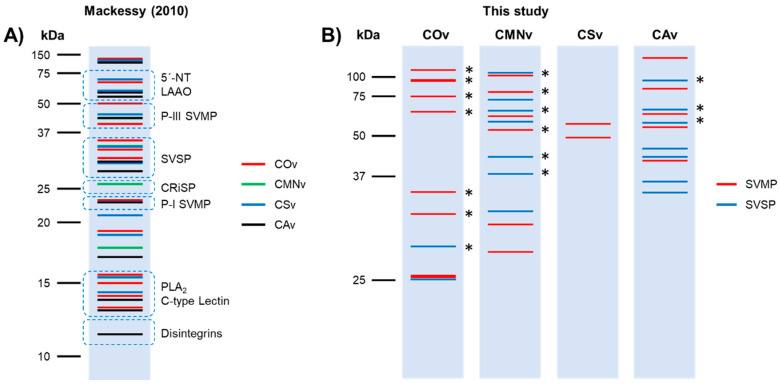
Schematic representation of SDS-PAGE and zymography bands observed in the venoms. (**A**) Summary of the bands observed in the SDS-PAGE for *C. ornatus* (COv, red line), *C. m. nigrescens* (CMNv, green line), *C. scutulatus* (CSv, blue line), and *C. atrox* (CAv, black line). Blue ovals enclose the typical molecular weight intervals for the most abundant toxin families according to Mackessy [53]. (**B**) Schematic representation of the SVMP (red line) and SVSP (blue lines) bands observed in zymographies for *C. ornatus* (COv), *C. m. nigrescens* (CMNv), *C. scutulatus* (CSv), and *C. atrox* (CAv). SVMP are represented in red lines and SVSP in blue lines. Asterisks denotes the proteolytic bands that have never been described in the literature.

Overall, all the venoms in this study have protein patterns characteristic of type I venoms, which are characterized by a high protease concentration and are thus predominantly fibrinogenolytic, fibrinolytic, and hemorrhagic venoms [53]. The summarization of the bands observed in our venom samples are shown in Figure 8A.

### 3.2. In-Gel Zymography

In-gel zymography is a technique used to demonstrate the molecular weight of hydrolytic enzymes [72]. Gelatin and casein were chosen to be used in zymography due to their universal use in this technique, and also by their structural characteristics, gelatin being a degradation product of collagen [73] and casein a globular protein.

Further, gelatin zymography has been used for a wide range of proteases, such as metalloproteases, MMPs, cysteine proteases, and serine proteases [74,75,76]. Casein zymography has been used mostly to screen serine proteases, cysteine proteases, and MMP-3 [77,78,79]. Contrary to what is described above, our experimentation failed to demonstrate a differential protease activity on gelatin and casein zymographies, observing a limited number of bands on casein zymography, in the case of *C. m. nigrescens*, *C. scutulatus,* and *C. atrox* venoms. The band diminishing on casein gel is also described for other snake venoms such as *Bothriechis schlegelii* [80], *Bothrops insularis* [81], and *Pseudoboa neuwieddi* [82].

In general, we found active bands within the molecular weight of the different types of SVMPs (including P-I, P-II, and P-III subtypes) and SVSPs in all our venom samples [83]. From our samples, *C. ornatus* venom demonstrated a higher P-I SVMP activity (23–28 kDa, Figure 3A,B) than that of the other venom samples and, in *C. scutulatus* and *C. atrox* venom samples, the activity was almost undetectable. On the other hand, *C. m. nigrescens* and *C. atrox* venoms demonstrated higher P-III compared to that of the other samples. Finally, only the *C. scutulatus* sample did not show SVSP activity.

From zymographies, several bands detected in both substrates were not detected in SDS-PAGE. This phenomenon can be explained due to the sensibility of both techniques, whereas Coomassie colloidal staining can detect 1 ng/band of protein [84], zymography have a detection limit as low as 10 pg/band [76], allowing the detection of toxins contained in the venom that have a low concentration. In addition, these venoms were tested for gelatin cleavage, a collagen degradation product. It could be hypothesized that the degradation of collagens was by SVMPs of these species, which is supported for *C. m. nigrescens* [62], *C. scutulatus* [68], and *C. atrox* venom samples [85,86]. Furthermore, for the potential application to wound healing P-I SVMP may be preferred over P-III SVMP for the hydrolyzation of collagen as P-III SVMP is more hemorrhagic and induces systemic bleeding [87]. Thus, due to its high P-I SVMP activity, *C. ornatus* venom could be a potential source of gelatinase-like MMP for use in impaired wound healing. Finally, our study demonstrated the presence of several SVMP and SVSP bands that have never been described in the literature (Figure 8B), mostly from the black tailed rattlesnakes, *C. ornatus* and *C. m. nigrescens*.

### 3.3. Protease Substrate Preference

From all venoms, *C. ornatus* extract demonstrated the highest proteolytic activity among all the venom samples. This result agrees with the data published by other authors, where *C. ornatus* venom have the highest proteolytic and hemorrhagic activities among *Crotalus* venoms [88], including several Viperidae and Elapidae venoms [24]. Contrary to our results, Soto et al. [88] described that *C. scutulatus* venom lacks gelatinolytic activity. Also, a study performed by Roldán-Padrón et al. [89] demonstrated that the venom caseinolytic activity of *C. atrox* is higher than that of *C. m. nigrescens*.

We additionally demonstrated the capability of *Crotalus* venom to proteolyze gelatin, a degradation product of collagen. Adding to this, SVMP have been described to degrade collagens present in skin [32], as is described for MMPs, to lead the wound healing and skin remodeling [90,91]. For this reason, it can be hypothesized that *Crotalus* SVMPs have the potential to mimic MMPs activity on skin wound healing.

### 3.4. Serine Protease Activity

The snake venom esterase activity on L-TAME demonstrated that *C. ornatus* and *C. m. nigrescens* samples had higher activity in comparison to that of *C. scutulatus* and *C. atrox* samples, these results are supported by Soto et al. [24] who described that *C. ornatus venom* has higher protease activity on L-TAME in comparison to that of other *Crotalus* species. Nevertheless, the esterase activity on *C. ornatus* and *C. atrox* venoms is lower in comparation to that of other species such as *Bothrops* spp. [24,92,93], *Deinagkistrodon acutus*, *Naja naja* [24,94], *Trimesurus malabaricus,* and *Daboia russelli* [95,96]. Even with the poor esterase activity described for our *Crotalus* venoms, SVSPs from these venoms produced fibrin clots (Figure 6B).

### 3.5. Fibrinogenolytic Activity

Fibrinogenolysis is a toxic effect described for *Crotalus* spp. and is generated by SVMPs and SVSPs [33,97]. These assays were performed to demonstrate A) the capacity of the venoms to hydrolyze fibrinogen and B) the role of SVMPs and SVSPs during this process. As we described previously, all venoms were Aα- and Bβ-fibrinogenolytic (Figure 6A). This phenomenon is already described for viperids, including *C. atrox*, *C. scutulatus*, *C. molossus nigrescens,* and *C. ornatus* [63,98,99,100]. Most of the Aα-fibrinogenolytic activity of crude venoms could be the consequence of the SVMP activity, given that such proteases are in higher abundance in *Crotalus* venoms. Moreover, when 1,10-P samples were incubated with fibrinogen, the SVSPs caused coagulation, this phenomenon is only described for CSv and CAv [100,101].

In wound healing, clot formation by thrombin has a pivotal role, providing a provisional matrix to protect the wound from pathogens and to allow the cell migration to start the healing process [102]. Thrombin activity can be mimicked by SVSPs, suggesting its applicability to wound healing. This approach was already explored by Ferreira et al. [38], proposing a SVSP from *Crotalus durrisus terrificus* as a wound sealant. Nevertheless, even when our result suggests that SVSP produce clot formation, further studies on clot stability must be performed through thromboelastography tests [103].

### 3.6. Fibrinolytic Activity

A fibrinolytic assay was performed to demonstrate that SVMPs in *Crotalus* venom perform this activity. Some fibrinolytic SVMPs have been isolated and thir activity has been proven from *C. ornatus* [59,60], *C. m. nigrescens* [63,64], *C. scutulatus* [104], and *C. atrox* venoms [100,105]. Fibrinolytic SVMPs from these venoms could be applied as wound healing agents to mimic plasmin activity, allowing the transition of a fibrin-rich matrix to a collagen matrix [106]. Plasmin is a protease responsible for fibrin homeostasis via clot degradation, and when it is absent there is a severely impaired re-epithelialization [107]. Thus, fibrinolytic SVMPs could be useful to enhance skin re-epithelialization during wound healing.

In conclusion, we described the presence of SVMPs and SVSPs in *C. ornatus*, *C. m. nigrescens*, *C. scutulatus,* and *C. atrox* venom extracts. These snake venom proteases are capable of degrading casein, gelatin, fibrinogen, and fibrin. From all venoms, we found that *C. ornatus* had a higher P-I SVMP activity in comparation to that of the other venom samples, which is also one of the most proteolytic venoms across *Crotalus* spp.’ venoms. In terms of their potential application to skin wound healing, we demonstrate that SVMPs can cause gelatin, casein, fibrinogen, and fibrin degradation and SVSPs generate clot formation through fibrinogenolysis. In other words, SVMPs could mimic the MMPs degradation of gelatin (collagen) to induce cell migration and mimic plasmin, allowing the formation of a collagen-rich extracellular matrix; on the other hand, SVSPs could be used to mimic thrombin to close open wounds, allowing for the next steps of wound healing.

## 4. Materials and Methods

### 4.1. Snake Venom Samples and Quantification

*Crotalus atrox* (CAv), *C. m. nigrescens* (CMNv), and *C. s. scutulatus* (CSv) venom samples were obtained from specimens maintained in captivity at Universidad Autónoma de Querétaro Herpetarium (SEMARNAT permit number: INE/CITES/DGVS-CR-IN-0619-QRO00). *Crotalus ornatus* venom (COv) was obtained from specimens maintained in captivity at Laboratorio de Ecología y Biodiversidad Animal from Universidad Autónoma de Ciudad Juárez (SEMARNAT permit number: SGPA/DGVS/04134/11). Venom extraction was performed as described previously [56]. After extraction, venom was pooled, lyophilized, and stored at −20 °C. First, 10 µg of lyophilized venoms were solubilized in 100 μL H_2_O, centrifuged for 15 min at 13,500 rpm using a Prism R centrifuge (Labnet, Edison, NJ, USA) in order to remove insoluble proteins and cellular debris. Total protein concentration of all venoms was determined using a Bradford protein assay using bovine serum albumin as the standard [108]. Briefly, 10 µL of each venom sample were mixed with 250 µL of Bradford reagent (Sigma-Aldrich, Saint Louis, MO, USA) and incubated 10 min. Absorbance was measured at 595 nm using a microplate reader (Synergy HT, Biotek, Winooski, VT, USA). Quantification was performed for three technical replicates.

### 4.2. SDS-PAGE 

Protein patterns from all venoms were observed in 12% SDS-PAGE according to Sambrook [109] and stained with Coomassie colloidal stain [84]. We used Image Lab 6.0.1 (BioRad, Hercules, CA, USA) to process apparent molecular weight for the bands in SDS-PAGE and zymography using 5 µL Precision Plus Protein™ Dual Xtra (BioRad, Hercules, CA, USA) as the molecular weight marker.

### 4.3. Proteolytic Activity 

For protease activity characterization, we chose gelatin and casein as substrates because of their universal use in these methods. Moreover, gelatin (a collagen derivative) was selected due to collagen degradation having a key role in wound healing [11].

Proteolytic activity was quantified using a previously reported method [110] with slight modifications. Briefly, 10 µg of each venom sample where diluted in 100 µL of PBS and incubated with 200 µL of 1% (*w/v*) casein solution (20 mM Tris-HCl, pH 7.4) for 1 h at 37 °C. The reaction was stopped with 15% (*w/v*) ice-cold trichloroacetic acid, and then the sample was centrifuged for 15 min at 2000 rpm using a Prism R centrifuge (Labnet, Edison, NJ, USA). Supernatant was collected and soluble digested protein was determined by a ninhydrin-based protein assay [42] using L-Leucine (Sigma-Aldrich, Saint Louis, MO, USA) as the standard. The results were expressed as specific protease activity units, mmol equivalent of L-leucine formed per minute per mg of protein. For the in-gel zymography experiments, 8 µg of each venom sample was mixed with a 4X non-reducing SDS-PAGE sample buffer (150 mM Tris-HCl, pH 6.8, 12% SDS, 30% glycerol, 0.005% bromophenol blue) and loaded on a 10% SDS-PAGE co-polymerized with 1% (*w/v*) gelatin or 1% (*w/v*) casein. Electrophoresis was carried at 90 V and 4 °C as previously described by Subramanian et al. [111]. Gels then were washed twice with washing buffer (50 mM Tris-HCl, pH 7.5, 5 mM CaCl_2_, 1 μM ZnCl_2_, 2.5% Triton X-100) for 30 min at room temperature and incubated with buffer (50 mM Tris-HCl, pH 7.5, 5 mM CaCl_2_, 1 μM ZnCl_2_, 1% Triton X-100) for 16 h at 37 °C. Zymograms were stained using Coomassie colloidal stain, and hydrolytic bands were visualized as a clear band on blue background.

In order to identify the protease families present in snake venoms in protease activity quantification, 10 µg of venom samples were independently incubated with 5 mM of ethylenediaminetetraacetic acid (EDTA), 1,10-Phenanthroline (1,10-P), phenylmethanesulfonyl fluoride (PMSF), and benzamidine hydrochloride hydrate (BA) for 10 min at 37 °C before substrate addition. Then, protease activity was performed with 1% (*w/v*) casein as substrate. The activity was expressed as relative activity from control as previously reported [112]. For substrate preference tests, 1% (*w/v*) substrate solutions were incubated with the venom samples as is described above, using as the substrate casein, gelatin, or hemoglobin. In order to identify protease families on zymography, gels were washed and incubated with the buffers added with 5 mM of EDTA, 1,10-P, PMSF, and BA.

### 4.4. Serine Protease Activity 

Assays to evaluate the serine protease activity were performed using *N*_α_-*p*-Tosyl-L-arginine methyl ester hydrochloride (L-TAME), according to the modified method described by Zheng et al. [113]. Briefly, 5 µg of all venoms were diluted in 75 µL of 10 mM Tris-HCl, pH 7.4, and incubated with 150 µL of substrate solution (1 mM L-TAME in 10 mM Tris-HCl, pH 7.4). The serine protease activity was quantified by spectrophotometric analysis at 247 nm for 15 min. The results were expressed as specific serine protease activity, as the increase of 0.01 units in absorbance per min per mg of protein.

For inhibition assays, venom samples were incubated with the PMSF and BA as is described for the protease activity assay for 10 min after substrate addition. Then, the assay was carried out as is described above. The activity was expressed as relative activity compared to the control.

### 4.5. Fibrinogenolytic Activity

Clot formation has a key role in wound healing. Thus, we tested the ability of the venom samples to clot fibrinogen. This human fibrinogen was isolated as previously described [114]. Samples were collected in BD Vacutainer ^TM^-buffered EDTA tubes, centrifuged for 10 min at 3000 rpm, and the platelet rich plasma (PRP) was collected. The fibrinogen was precipitated from the PRP, adding 0.3 volumes of precipitation solution (50% (*w/v*) ammonium sulfate, 0.05% (*w/v*) sodium azide in PBS). Then, the PRP was washed thrice with 12% (*w/v*) ammonium sulfate in PBS and solubilized in PBS. Fibrinogen was stored at 4 °C until use.

Fibrinogenolytic activity was performed as previously described [114]. Briefly, 10 µg of all venoms diluted in 10 µL of 50 mM Tris-HCl solution (pH 7.4) were incubated with 20 µL of 5 mg/mL fibrinogen solution (50 mM Tris-HCl, pH 7.4) at 37 °C at different time intervals (0–180 min). The reactions were stopped using 6 µL of 4X loading buffer (150 mM Tris-HCl, pH 6.8, 12% SDS, 30% glycerol, 5% β-mercaptoethanol, 0.005% bromophenol blue) and analyzed in a 10% SDS-PAGE. Fibrinolytic activity was considered positive when Aα or Bβ fibrinogen peptides were degraded by the venoms.

Corresponding inhibition assays were performed by pre-incubating the venom samples with inhibitors for 10 min before substrate addition. The activity assay was carried out as is described above, incubating the venom extract-inhibitor mixture with fibrinogen for 60 min.

### 4.6. Fibrinolytic Activity

Fibrin clot degradation is needed during the wound healing process to allow for re-epithelialization. For this reason, we tested the ability of the venoms to degrade fibrin. The fibrinolytic assays were performed in fibrin-agarose plates as described previously [115]. Plates were prepared by mixing 1% agarose with 0.1% fibrinogen to a final volume of 10 mL. Two units of thrombin (100 µL) were added to induce fibrin coagulation and 10 µg of venom samples were added into 3 mm wells in the fibrin-agarose plate and incubated at 37 °C for 15 h. The results were expressed as fibrinolytic activity in mm of degraded fibrin in mm (clear fibrin area). For inhibition tests, venom samples were previously incubated with the same inhibitors as is described for the protease activity assay.

## Supplementary Materials

The following are available online, Figure S1: In-gel zymography optimization for snake venom protease screening.
