# Supplementary material for: Functional Mining of the Crotalus Spp. Venom Protease Repertoire Reveals Potential for Chronic Wound Therapeutics
Source: Molecules. 2020 Jul 28;25(15):3401. doi: 10.3390/molecules25153401 (PMC7435869; doi:10.3390/molecules25153401)
Supplement: Supplementary file 1 [file molecules-25-03401-s001.pdf]

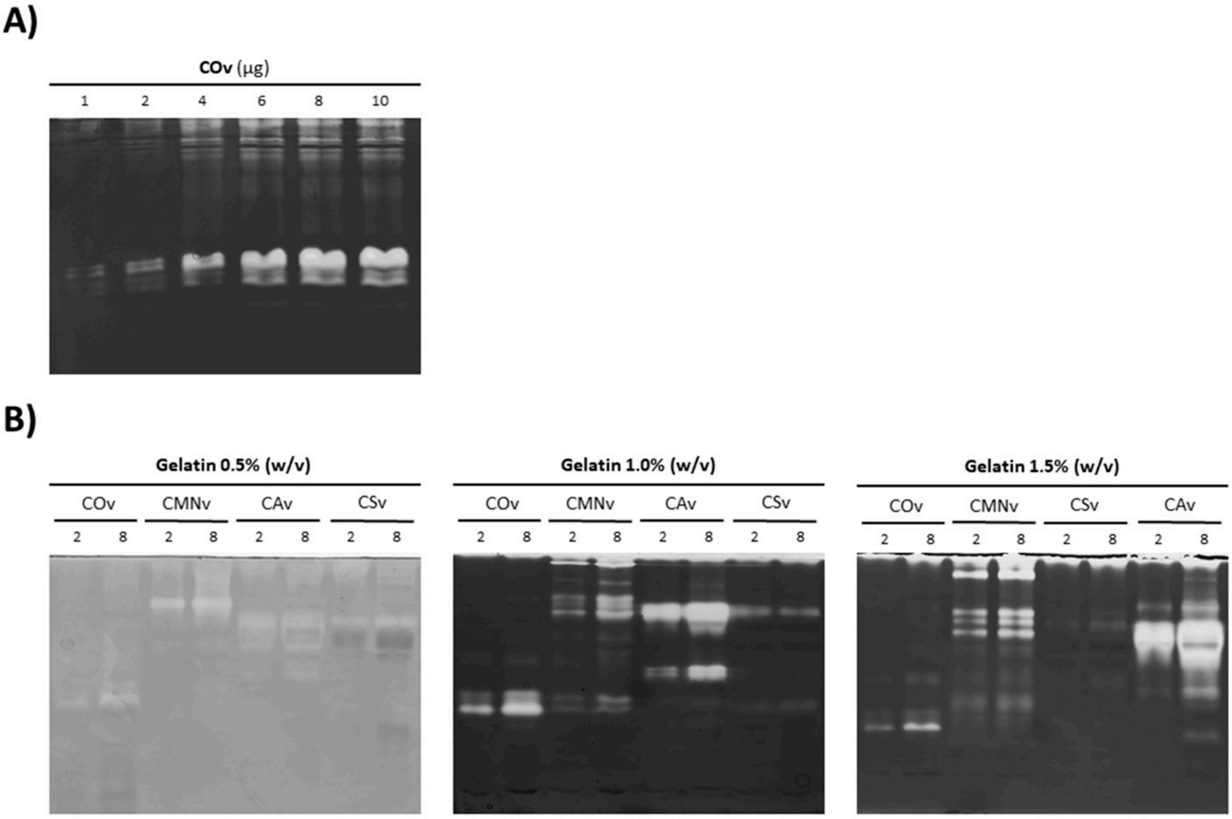

Figure S1. In-gel zymography optimization for snake venom protease screening. (**A**) Optimization of the venom sample concentration using *C. ornatus* venom (1, 2, 4, 6, 8 and 10 µg) on a 1.0% (w/v) gelatin zymography. (**B**) Optimization of the gelatin as substrate (0.5, 1.0 and 1.5% w/v) on the zymography using *C. ornatus* (COv), *C. m. nigrescens* (CMNv), *C. scutulatus* (CSv) and *C. atrox* (CAv) venoms.
